# Fractionation, Bioaccessibility, and Risk Assessment of Heavy Metals in the Soil of an Urban Recreational Area Amended with Composted Sewage Sludge

**DOI:** 10.3390/ijerph15040613

**Published:** 2018-03-28

**Authors:** Kai Yang, Tao Zhang, Yanqiu Shao, Chao Tian, Stephen R. Cattle, Ying Zhu, Jinjuan Song

**Affiliations:** 1 Advanced Materials Institute, Qilu University of Technology (Shandong Academy of Sciences), Jinan 250014, China; yangk@sdas.org (K.Y.); zhangtao@sdas.org (T.Z.); shaoyq@sdas.org (Y.S.); tianchao@sdas.org (C.T.); 2 College of Environmental Science and Engineering, Taiyuan University of Technology, Jinzhong 030600, China; 3 Sydney Institute of Agriculture, School of Life and Environmental Sciences, Faculty of Science, The University of Sydney, Eveleigh, NSW 2015, Australia; stephen.cattle@sydney.edu.au; 4 Quality, Safety and Environmental Protection Department, China International Water and Electric Corporation, Beijing 100120, China; song_jinjuan@ctg.com.cn

**Keywords:** biosolids, land application, trace elements, sequential extraction, in vitro test, mobility, exposure, factors

## Abstract

A composted sewage sludge (CSS) was added to the soil of an urban garden at 5%, 10%, and 25% (*w*/*w* soil) and stabilised for 180 days. Samples were then collected and analysed for total heavy metal concentrations, chemical fractions, and bioaccessibility, together with some physicochemical properties. The results showed that the total chromium (Cr), copper (Cu), lead (Pb), and zinc (Zn) concentrations were increased with CSS addition rate. The CSS addition decreased the residual fractions of these four elements. The exchangeable Cr, Cu, and Pb fractions were very small or not detected, while Zn exhibited an increasing trend in its exchangeable fraction with CSS addition rate. The bioaccessibility of these four elements was increased with the CSS addition rate. Moreover, the Cr, Cu, and Zn bioaccessibility correlated positively with the total concentration, while the bioaccessibility of these four elements exhibited a negative correlation with the residual fraction. The fractionation and bioaccessibility of heavy metals may have also been influenced by pH, cation exchange capacity, and organic matter. The risk assessment code reflected the amended soil showed no or low environmental risks for Cr, Cu, and Pb and a medium risk for Zn. The hazardous index values and cancer risk levels indicated that the heavy metals in the soil amended with 25% CSS posed negligible potential noncarcinogenic and carcinogenic risks to children and adults via incidental ingestion.

## 1. Introduction

Biosolids (treated sewage sludge (SS)) contain organic carbon (C), nitrogen (N), phosphorus (P), potassium (K), sulphur (S), calcium (Ca), magnesium (Mg), and microelements necessary for plants and soil fauna to live [1]. Land application of biosolids is a promising alternative of disposal worldwide as it offers the possibility of recycling plant nutrients and organic matter (OM) economically as well as possibly contributing to soil C sequestration [1,2]. According to the Asian Development Bank [3], there are 17.8 and 9.0 million tons of dry solids produced per year in the United States and European Union, respectively, with 40–55% of the biosolids applied to land. Australia has been recently estimated to produce 0.327 million tonnes of dry solids per year, with 94% of the biosolids used in agriculture (75%), land rehabilitation (11%), and landscaping (8%) [4]. In China, there were 6.25 million tons of dry solids produced in 2013, with more than 80% of the SS disposed by improper dumping [5]. Approximately 16.8 million tons of dry solids will be produced in China in 2020 [6]. Sanitary landfill persists as a predominant SS disposal method in China [7]. The application of SS in soils has been precluded largely due to the higher concentrations of heavy metals compared with soils, which can result in soil and water contamination and plant phytotoxicity as well as pose a potential threat to human health along the food chain [7,8].

Many studies have examined the accumulation and migration of heavy metals in the soil–plant system as affected by SS amendment. The increasing trend in concentrations of some heavy metals in plant tissues with SS addition rate and an inhibitive effect on plant growth due to excessive application have been recorded in the literature [9,10,11,12,13]. Some researchers have worked on the fractionation of heavy metals in SS and SS-amended soils to estimate their mobility and availability to plants [14,15,16,17,18]. For example, Bai et al. [14] observed that the increment of metal uptake by maize grown in the SS-amended mudflat saline soil was determined by the increment of available metal concentrations (viz. the acid-soluble/exchangeable fraction) in the soil. It has been suggested that using SS as a fertiliser in landscaping can avoid the potential transmission of heavy metals in SS through the food chain [11]. However, it should be noted that in playgrounds and other recreational areas, the incidental ingestion of soil via hand-to-mouth activity is a very likely way of transferring metals from SS-amended garden soils to humans, especially children [19,20]. To date, there has been limited work on the oral bioaccessibility of heavy metals in SS and SS-amended soils, which indicates metal toxicity in humans. Tongesayi et al. [20] reported that the practice of amending soils with regulated biosolids can present a real human health danger due to the accumulation of metals and the impact this will have on the speciation, mobility, and bioaccessibility of metals in the soil. Florido et al. [19] demonstrated that the addition of biosolids should be done only after making sure that the heavy metals present in the material are not likely to cause significant increases in the soil contents of these elements, especially the available and bioaccessible fractions. Moreover, research into the bioavailability of metals in biosolids applied to land has been suggested by the Environment Protection Authority of Victoria, Australia, to produce a more realistic estimation for risks associated with biosolids use in the current Victorian state guideline for biosolids land application [21].

The main aims of this study are therefore to: (a) investigate the effect of composted sewage sludge (CSS) addition on heavy metal accumulation, fractionation, and bioaccessibility as well as physicochemical properties in the soil of an urban recreational area; (b) explore the correlation between heavy metal fractionation and bioaccessibility in the amended soil and related influencing factors; and (c) estimate the environmental and health risks of heavy metals in the amended soil.

## 2. Materials and Methods

### 2.1. Soil and CSS

An approximately 10-kg composite soil sample, consisting of 10 random subsamples, was collected from the 0–0.1 m surface layer of an existing typical recreational garden (approximately 0.35 ha, N 36°38′38.66″, E 117°2′29.69″) in the Shandong Academy of Sciences in Jinan, using a plastic trowel. The CSS was supplied by Guangdong Huayang Environmental Science and Technology Co., Ltd., in Zhaoqing, China. The CSS has been used in commercial tree planting to supply nutrients and OM for years. The soil and CSS were air-dried, gently crushed, and passed through a 2-mm stainless steel sieve to remove gravels, plant materials, and other debris.

### 2.2. Incubation Test

The CSS was mixed homogeneously with the soil at three doses, viz. 5%, 10%, and 25% (*w*/*w* soil). Approximately 500 g of the mixture was then packed into a plastic pot (120 mm in height, 80 mm in diameter) with air holes on its top cap. Unamended soil was parallelly prepared as control. Each treatment had three replicates, with a total of 12 pots. All mixtures and control were incubated under aerobic conditions at 25 °C in an incubator for 180 days, keeping a soil moisture content of 18% using deionised water by weighting. After a 180-day ageing, the sample in each pot was mixed thoroughly, air-dried, and gently crushed for subsequent analyses. There were 12 samples collected in total.

### 2.3. Physicochemical Analysis

Sample pH and electrical conductivity (EC) were determined by the 1:5 solid/water (*w*/*v*) suspension method using a calibrated pH meter (PHS-3E, Rex, Shanghai, China) and a EC meter (DDS-307A, Rex, Shanghai, China) [22]. Cation exchange capacity (CEC) was determined by the ammonium acetate titration method [22]. OM content was determined by the potassium dichromate titration method [22]. Total N (TN) content was determined by the Kjeldahl method [22]. Particle size distribution was determined by the laser diffraction method using a particle size analyser (Mastersizer 2000, Malvern, Malvern, UK) [23].

### 2.4. Heavy Metal Analysis

Heavy metals including chromium (Cr), copper (Cu), lead (Pb), and zinc (Zn) were selected as elements-of-interest because they were among those having relatively high concentrations in the CSS as shown in our preliminary survey using a portable X-ray fluorescence (pXRF) analyser (DELTA Handheld DPO4050, Olympus, Waltham, MA, USA). Total heavy metal concentrations were determined by first digesting the sample using the hydrofluoric acid–perchloric acid–nitric acid (HF–HClO_4_–HNO_3_) solution method [22]. The acid digestion suspension was then filtered through a 0.45-μm syringe filter.

Heavy metal fractionation was determined by the modified Community Bureau of Reference (BCR) sequential extraction procedure [24]. The three-step method divides metals into four chemical fractions, viz. F1: exchangeable fraction; F2: reducible fraction; F3: oxidisable fraction; and F4: residual fraction.

Step 1 (F1): 1 g of sample was added to 40 mL of 0.11 mol/L acetic acid solution in a 100-mL centrifuge tube, and shaken for 16 h on a reciprocal shaker at 22 ± 5 °C. The extract was then separated from the solid residue by centrifugation at 3000× *g* for 20 min and filtered through a 0.45-μm syringe filter. The residue was washed by adding 20 mL of deionised water, shaken for 15 min, and centrifuged at 3000× *g* for 20 min.

Step 2 (F2): the residue from Step 1 was added to 40 mL of 0.50 mol/L hydroxylammonium chloride (adjusted to pH 2.0 ± 0.1 with concentrated nitric acid) and shaken for 16 h at 22 ± 5 °C. The extract was then separated from the solid residue by centrifugation and filtration as in Step 1. The residue was washed as in Step 1.

Step 3 (F3): the residual from Step 2 was added to 10 mL of 30% hydrogen peroxide (adjusted to pH 2.0 ± 0.1 with concentrated nitric acid), and digested for 1 h at 22 ± 5 °C. The digestion was continued for 1 h at 85 ± 2 °C. A further aliquot of 10 mL of 30% hydrogen peroxide (pH 2.0 ± 0.1) was added and kept digesting for 1 h at 85 ± 2 °C. 50 mL of 1.0 mol/L ammonium acetate (adjusted to pH 2.0 ± 0.1 with concentrated nitric acid) was added to the cool moist residue and shaken for 16 h at 22 ± 5 °C. The extract was then separated from the solid residue by centrifugation and filtration as in Step 1.

Step 4 (F4): the residue from Step 3 was analysed as per the same procedures in the total heavy metal concentration analysis.

Heavy metal bioaccessibility was determined by the simplified physiologically-based extraction test (SBET) [25,26]. 1 g of sieved sample (<250 μm) was added to 100 mL of a simulated gastric solution (0.4 M glycine adjusted to pH 1.50 ± 0.05 with concentrated hydrochloric acid) in a 125-mL wide-mouth high-density polyethylene (HDPE) bottle, and shaken for 1 h on a gas bath reciprocal shaker at 37 ± 2 °C. The suspension was then filtered through a 0.45-μm syringe filter. The final pH of the suspension should remain within ±0.5 pH units of the initial pH, otherwise the procedure has to be redone. The fraction of samples of <250 μm was used for bioaccessibility testing, as it is considered to be the most representative of what adheres to children’s hands and most likely to be ingested [26]. Metal bioaccessibility is expressed as a percentage of bioaccessible concentration to total concentration.

All above filtrates were measured to determine metal concentrations using inductively coupled plasma-atomic emission spectrometry (ICP-AES) (Optima 7000 DV, PerkinElmer, Waltham, MA, USA) at Shandong Analysis and Test Center in Jinan. Quality control for these digestion/extraction tests was assessed by the use of a blank, Chinese National Standard Reference Material (SRM) (GBW07449 (GSS-20)) saline–alkali soil, National Institute of Standards and Technology (NIST) SRM 2711a Montana soil II, and duplicate samples. The recovery rates for all selected elements in the SRMs were within 100 ± 10%. The relative standard deviations between the duplicate samples were ≤5%.

### 2.5. Environmental and Health Risk Assessments

To assess the environmental risk of heavy metals in the amended soils, the risk assessment code (RAC), defined as the fraction of metal exchangeable and/or associated with carbonates (F1 %), was determined for Cr, Cu, Pb, and Zn, and the values were interpreted in accordance with the RAC classifications [27]. If RAC is <1%, the soil is of no risk to the environment. Low risk, medium risk, high risk, and very high risk are associated with RAC values of 1–10%, 11–30%, 31–50%, and >50%, respectively.

The health risks from exposure to heavy metals in the amended soils via hand-to-mouth activity were estimated by the hazard index method and cancer risk method [28]. As Cr exhibits strong carcinogenic potential in humans [29], the carcinogenic risk for Cr was calculated. The noncarcinogenic risks for Cr, Cu, Pb, and Zn were calculated. For exposure assessment, the chronic daily intake (*CDI*) of individual metals from incidental ingestion of soil was calculated using the following equation [28]:

(1) $$CDI=C\times \frac{I_{R}\times E_{F}\times E_{D}}{W_{AB}\times T_{A}}.$$

where *CDI* is the chronic daily intake (mg/(kg·day)); *C* is the exposure concentration of the soil metal (mg/kg); *I_R_* is the ingestion rate at 200 mg soil/day for children and 100 mg soil/day for adults; *E_F_* is the exposure frequency of 180 days/year; *E_D_* is the exposure duration of 6 years for children and 24 years for adults; *W_AB_* is the average body weight of 15 kg for children and 70 kg for adults; and *T_A_* is the average time (for noncarcinogens, *T_A_* = *E_D_*; for carcinogens, *T_A_* = 70 years). The exposure parameter values were referred to the study of Hu et al. [25], who employed the same methods to assess the health risks of heavy metals in street dusts for children and adults via incidental ingestion in China.

The noncarcinogenic and carcinogenic risks for individual metals were calculated using the following equations [28]:

(2) $$\mathrm{Hazard}\;\mathrm{quotient}=\left(CDI\times OBA\right)/R_{f}D_{o}$$

(3) $$\mathrm{Carcinogenic}\;\mathrm{risk}=CDI\times OBA\times SF_{o}$$

where *OBA* is the oral bioaccessibility of the soil metal; *R_f_D_o_* is the reference dose (0.003 mg/(kg·day) for Cr, 0.04 mg/(kg·day) for Cu, 0.0035 mg/(kg·day) for Pb, and 0.3 mg/(kg·day) for Zn); and *SF_o_* is the slope factor (0.5 (kg·day)/mg for Cr).

Previous studies ([30] and references therein) reported that exposure to two or more pollutants may result in additive and/or interactive effects. Hazard quotients (HQs) can therefore be summed across constituents to generate the hazard index (HI) for a specific receptor/pathway (e.g., soil ingestion) combination. In this study, the HI is a measure of the potential risk of noncarcinogenic effects from a mixture of Cr, Cu, Pb, and Zn in the amended soils via incidental ingestion. According to the United States Environmental Protection Agency [28], if the value of HI is less than one, it is believed that there is no significant risk of noncarcinogenic effects. If HI exceeds one, then there is a chance that noncarcinogenic effects may occur, with a probability which tends to increase as the value of HI increases. Cancer risks lower than 1 × 10^−4^ are generally considered to be acceptable, while risks above 1 × 10^−4^ are regarded to be sufficiently large that some sort of remediation is desirable.

### 2.6. Data Analysis

For each variable, data were presented as the mean ± standard deviation of three independent replicates. Statistical analysis was performed using IBM^®^ SPSS^®^ Statistics 20 (Armonk, NY, USA). The normality of distribution was checked by the Kolmogorov–Smirnov test and normal Q–Q plot. Variables which were not normally distributed were then log_10_-tranformed. The differences between doses were analysed using one-way analysis of variance (ANOVA). The homogeneity of variances was checked by Levene’s test, and the means were compared by the least significant difference (LSD) test at a significance level of *p* < 0.05. To identify the factors influencing bioaccessibility, correlations between various variables were determined using Pearson’s product moment correlation coefficient (*r*).

## 3. Results and Discussion

### 3.1. Physicochemical Properties

The physicochemical properties of the garden soil and CSS are shown in Table 1. The soil was moderately alkaline (pH 8.46), while the CSS was slightly alkaline (pH 7.58). Moreover, the soil pH was close to the pH of the main urban park soils in Jinan (Table 1). The EC, CEC, and OM content of the CSS were conspicuously higher than of the soil. The high EC value (5013 μS/cm) of the CSS indicated that only a few very tolerant plants would yield satisfactorily, while the low EC value (142 μS/cm) of the soil indicated a negligible salinity effect on plant growth. The high CEC value (45.4 cmol(+)/kg) of the CSS suggested a high capacity for nutrient retention, while the lower CEC value (15.3 cmol(+)/kg) of the soil suggested a medium capacity for nutrient retention. The soil had much lower OM content (0.64%) and TN content (0.04%) than the CSS (25.63% and 1.20%, respectively). In addition, the low OM and TN contents of the soil were consistent with the generally low soil fertility of the main urban parks in Jinan (Table 1). According to the international soil texture classification system recommended by the International Society of Soil Science (ISSS) [31], the soil was classified as a silty loam, and the texture of the CSS was similar to that of silty loam.

**Figure 1:**
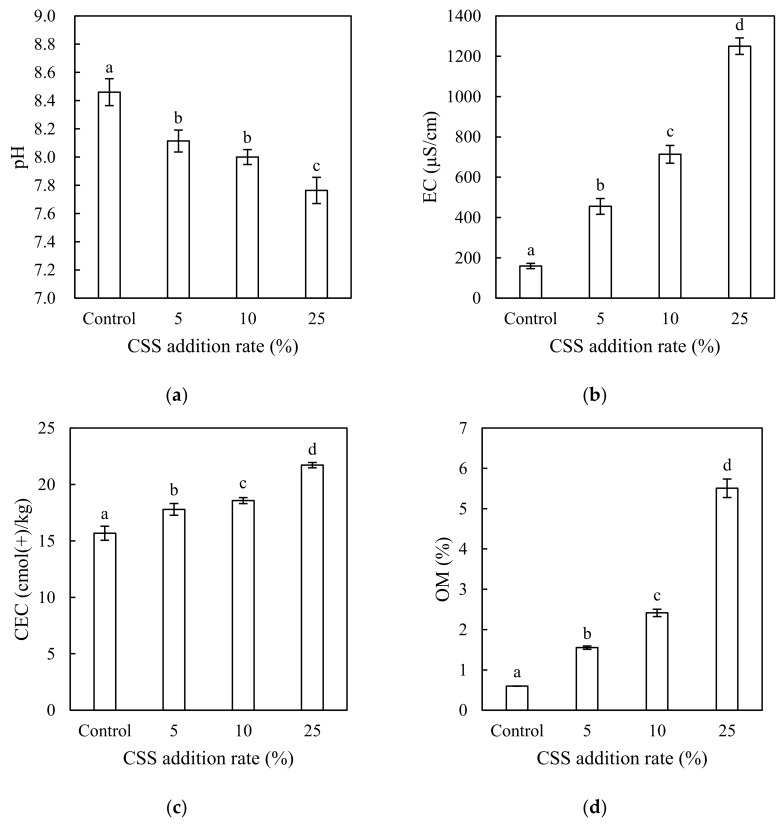
(**a**) pH of the control and amended soil; (**b**) electrical conductivity (EC) of the control and amended soil; (**c**) cation exchange capacity (CEC) of the control and amended soil; (**d**) organic matter (OM) of the control and amended soil. For a given property, the columns labelled with the same letter were not significantly different at a significance level of *p* < 0.05 by the least significant difference (LSD) test. The error bars represent one standard deviation (*n* = 3). CSS: composted sewage sludge.

The effect of CSS addition on the pH, EC, CEC, and OM content of the amended soil are shown in Figure 1. The pH was decreased significantly with CSS addition rate, from moderately alkaline (pH 8.46) in the control to slightly alkaline (pH 7.76) in the soil amended with 25% CSS. Compared with the garden soil, the CSS had a much higher OM content. The obvious reduction in the pH can be attributed to the hydrolysis of CSS-originated OM caused by acid-producing bacteria producing organic acid during the incubation period [7,19]. On the contrary, there was a significant increasing trend in the EC, CEC, and OM content of the soil with CSS addition rate. The EC was increased from 159 μS/cm in the control to 1250 μS/cm in the soil amended with 25% CSS. The prominent increase in the EC is not surprising, as the CSS was sourced from municipal refuse and contained a large amount of soluble salts. However, it should be noted that the addition of CSS at a high rate (e.g., 25%) could result in excessively high salinity which may restrict the yields of many plants. The increasing trend in the EC of the CSS-amended soils with CSS addition rate was also observed previously [10,19]. Cheng et al. [10] further demonstrated that the addition of CSS at a rate of >20% caused an inhibition effect on the seedling emergence of perennial ryegrass, primarily due to the presence of excessive soluble salts. The CEC was increased from 15.7 cmol(+)/kg in the control to 21.7 cmol(+)/kg in the soil amended with 25% CSS. The OM content was increased from 0.60% in the control to 5.51% in the soil amended with 25% CSS. The changes in the CEC and OM content agree with those expected from the addition of the OM-rich CSS with a relatively high CEC, indicating a positive effect on soil fertility.

### 3.2. Total Heavy Metal Concentrations

The total Cr, Cu, Pb, and Zn concentrations in the CSS were 138.5 ± 3.5, 280.0 ± 15.6, 63.9 ± 1.6, and 337.0 ± 5.7 mg/kg; approximately 2.0-, 12-, 2.2- and 4.6-times higher than in the garden soil, respectively (Table 2). Table 2 shows the effect of CSS addition on total heavy metal concentrations. The total Cr, Cu, Pb, and Zn concentrations in the soil were increased significantly with CSS addition rate. This increasing trend is not surprising given that the total concentrations of these four elements in the CSS were higher than in the garden soil. According to the current national quality standard for SS used in landscaping (GB/T 23486–2009) in China (Table 3), the total Cr, Cu, Pb, and Zn concentrations in the CSS were all below the ceiling concentrations. The total Cr, Cu, Pb, and Zn concentrations in the garden soil were all below the ceiling concentrations of Grade A in the current national soil quality standard (GB 15618–1995) in China (Table 3). Moreover, the total concentrations of these four elements in the garden soil were close to the soil background values in Shandong Province (Table 3). However, all amended soils were classified as Grade B, with the total Cu, Pb, and Zn concentrations exceeding the ceiling concentrations of Grade A. This finding suggests that the addition of CSS resulted in a deterioration of the garden soil quality. Additionally, compared with the Australian health investigation levels (HILs) for soil contaminants (Table 3), the total concentrations of the selected heavy metals in the garden soil were all below the HIL A (residential) values. The CSS addition did not result in the total concentrations of these four elements exceeding the HIL A values, and the total concentrations of these four elements in all amended soils were significantly below the HIL C (recreational) values.

### 3.3. Fractionation and Bioaccessibility of Heavy Metals

**Figure 2:**
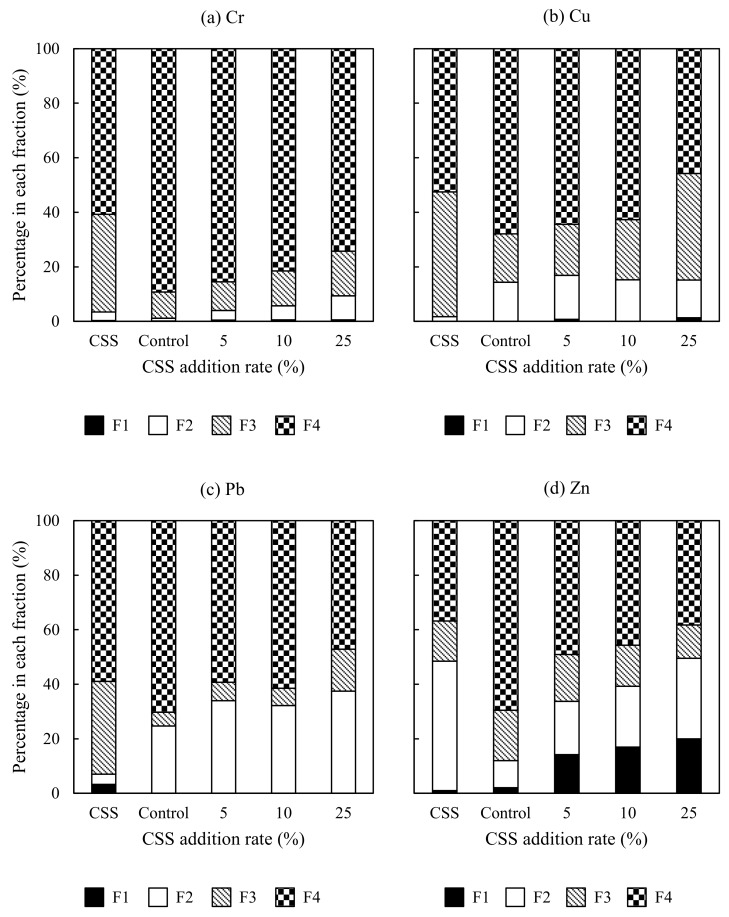
Heavy metal fractionation in the CSS, control, and amended soil. F1: exchangeable fraction; F2: reducible fraction; F3: oxidisable fraction; F4: residual fraction. (**a**) Cr fractionation in the CSS, control, and amended soil; (**b**) Cu fractionation in the CSS, control, and amended soil; (**c**) Pb fractionation in the CSS, control, and amended soil; (**d**) Zn fractionation in the CSS, control, and amended soil.

Figure 2 shows the effect of CSS addition on heavy metal fractionation. For Cr, 60.8% of Cr existed in F4 in the CSS, followed by 35.8% in F3. Cr was predominated by F4 in the control and amended soil, with a decreasing trend with CSS addition rate. The percentage of Cr in F4 was decreased from 89.3% in the control to 74.2% in the soil amended with 25% CSS. The oxidisable Cr predominated the nonresidual fractions in the control and amended soil, while a very small percentage of Cr was extracted in F1.

Regarding Cu, 52.6% of Cu was bound to F4 in the CSS, followed by 45.7% in F3. The distribution of Cu was similar to that of Cr, with a smaller percentage of Cu in F4 than Cr. The percentage of Cu in F4 was decreased from 67.9% in the control to 45.8% in the soil amended with 25% CSS. The addition of CSS at a rate of 25% resulted in a pronounced increase in the percentage of Cu in F3. This is probably due to the known much-higher affinity of Cu for OM and highly increased CEC and OM contents of the slightly alkaline amended soil [37]. Many studies reported that Cu was highly associated with the oxidisable fraction in the biosolids and biosolids/SS-amended soils [17,18,20]. There was only a very small percentage of exchangeable Cu in the soil amended with 25% CSS.

With respect to Pb, 59.0% of Pb was present in F4 in the CSS, followed by 34.0% in F3, 3.7% in F2, and 3.2% in F1. Pb was mainly bound to F4 in the control and amended soil, with a generally decreasing trend with CSS addition rate. The percentage of Pb in F4 was decreased from 70.3% in the control to 47.1% in the soil amended with 25% CSS. In contrast to Cu, the reducible Pb was the predominant fraction among the nonresidual fractions in the control and amended soil, indicating that the iron (Fe)–manganese (Mn) oxides may have played a significant role in binding Pb. This finding is consistent with the study of Shrivastava and Banerjee [18], who found the highest percentage of Pb to be associated with the reducible fraction among the nonresidual fractions in the SS-amended soil. Pb is known for its strong association with OM [37]. OM also influenced Pb retention in the amended soil, given a relatively high percentage of oxidisable Pb in the soil amended with 25% CSS with an OM content of 5.51%. There was no exchangeable Pb detected in the control and amended soil.

In the case of Zn, unlike the distribution of Cr, Cu, and Pb, Zn predominated in F2 in the CSS, with 36.8% in F4 and 14.8% in F3. Tongesayi et al. [20] also found Zn to be highly associated with the reducible fraction of biosolids. Zn was primarily extracted in F4 in the control. Both the percentages of Zn in F3 and F4 were decreased with CSS addition rate, while both the percentages of Zn in F1 and F2 were increased with CSS addition rate. The percentage of Zn in F4 was decreased to 38.3% in the soil amended with 25% CSS. Compared with Cr, Cu, and Pb, a notably large amount of exchangeable Zn was extracted in the amended soil. The percentage of Zn in F1 was increased from 2.1% in the control to 20.0% in the soil amended with 25% CSS. This can be attributed to the transformation of reducible Zn sourced from the CSS to exchangeable Zn at lower pH [38]. Qiao et al. [17] also demonstrated the sensitivity of Zn to soil acidity.

Having more metals associated with the nonresidual fractions will increase the potential of the metals’ mobility in the SS and SS-amended soils [18]. The potential mobility of these four elements in the CSS, control, and amended soil was in the following order: Zn > Cu > Pb > Cr. Some other researchers also found that Zn was the most mobile element whereas Cr was the least mobile in the SS and SS-amended soils [15,18]. Overall, the addition of CSS increased the potential mobility of Cr, Cu, Pb, and Zn to varying degrees, and the effect became more obvious with CSS addition rate. This can be ascribed to the following two possible reasons: on one hand, the potential mobility of these four elements in the CSS was higher than in the control; on the other hand, the addition of CSS changed the soil physicochemical properties such as pH and OM content, which can influence metal retention by the amended soil.

**Figure 3:**
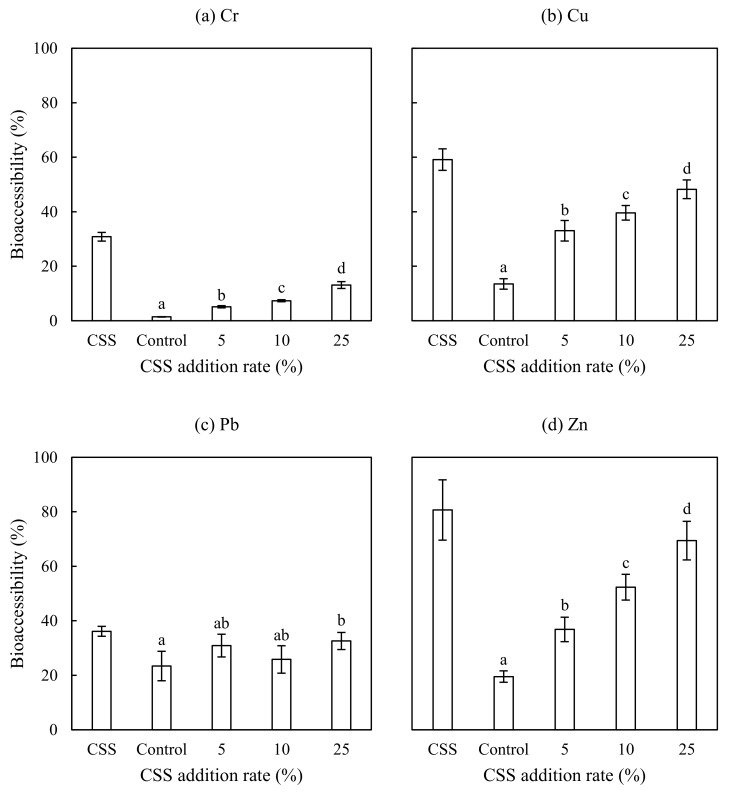
Heavy metal bioaccessibility in the CSS, control, and amended soil. For a given element, the columns labelled with the same letter were not significantly different at a significance level of *p* < 0.05 by the LSD test. The error bars represent one standard deviation (*n* = 3). (**a**) Cr bioaccessibility in the CSS, control, and amended soil; (**b**) Cu bioaccessibility in the CSS, control, and amended soil; (**c**) Pb bioaccessibility in the CSS, control, and amended soil; (**d**) Zn bioaccessibility in the CSS, control, and amended soil.

Figure 3 shows the effect of CSS addition on heavy metal bioaccessibility. The Cr, Cu, Pb, and Zn bioaccessibility was 30.8%, 59.1%, 36.1%, and 80.7% in the CSS, respectively, much higher than in the control (viz. 1.4% for Cr, 13.5% for Cu, 23.4% for Pb, and 19.5% for Zn). The Cr, Cu, and Zn bioaccessibility was increased significantly with CSS addition rate. The Pb bioaccessibility also showed some increases with CSS addition rate, but in this case the differences were not significant, except for a significant increase in the Pb bioaccessibility in the soil amended with 25% CSS compared with the control. The bioaccessibility of these four elements in the CSS, control, and amended soil was in the following order: Zn > Cu > Pb > Cr.

Table 4 lists the correlation coefficients (*r*) between the heavy metal bioaccessibility and various variables. In the case of Cr, Cu, and Zn, the bioaccessibility showed significant positive correlations with the corresponding total concentration, EC, CEC, and OM content, and significant negative correlations with the corresponding residual fraction and pH, respectively. Regarding Pb, its bioaccessibility only showed a significant negative correlation with the residual fraction. Many studies have found that metal(loid) bioaccessibility was closely related to the total concentration in the soil [19,20,25,39,40,41]. This agrees with the positive correlation between the metal bioaccessibility and total concentration in this study. The negative correlation between the metal bioaccessibility and residual fraction is consistent with the studies of Liu et al. [42] and Pascaud et al. [43], who demonstrated that metal(loids) associated with the residual fraction was difficult to be digested by the mimic human gastric fluid. Metal(loid) bioaccessibility can also be affected by a variety of soil physicochemical properties [19,25,39,40,41,44,45,46]. Soil pH is a key factor controlling metal availability, as an increase in pH increases the soil surface negative charge density and decreases the amount of H^+^ ions available to compete with metal ions for binding sites [39]. This is in agreement with the negative correlation between the metal bioaccessibility and pH in this study. OM has been well-known to bind metals strongly due to a large number of negative charges on its surface [37]. The positive correlation between the bioaccessibility and OM content for Cr, Cu, and Zn in this study suggested that the complexes of these three elements formed with OM were not stable in the gastric phase. Florido et al. [19] observed a similar significant correlation between the gastric metal bioaccessibility and OM content in the public park soils amended with composted biosolids. However, previous studies have demonstrated an inconsistent correlation between metal bioaccessibility and OM content in soils. For example, Liu et al. [42] reported that Pb bound to sulphides and OM did not significantly contribute to the gastric bioaccessibility of Pb in mining-impacted soils and dusts. Both Patinha et al. [47] and Yang and Cattle [41] noticed a positive correlation between the Pb bioaccessibility and OM content in mining-impacted soils. Conversely, Cai et al. [44] observed a beneficial effect of OM on reducing the gastric bioaccessibility of barium (Ba), Cu, and Pb, with less clear effects on Zn in the urban garden and orchard soils. CEC can enhance the chemisorption of metals by the soil at higher levels. The positive correlation between the metal bioaccessibility and CEC in this study can be attributed to the formation of soluble metal complexes with OM.

### 3.4. Environmental and Health Risk Assessment

**Figure 4:**
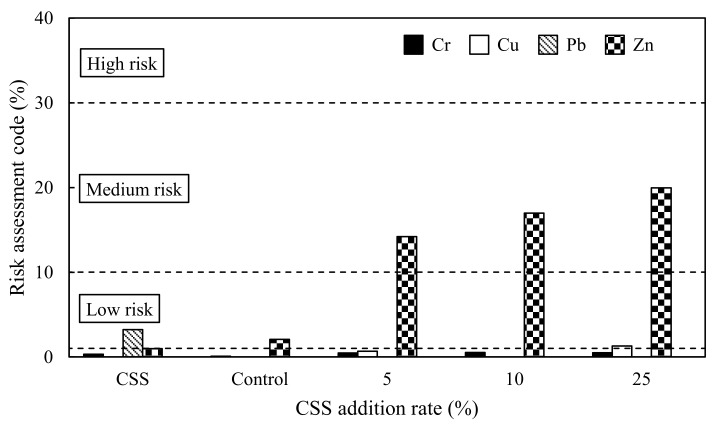
Risk assessment code (RAC) for heavy metals in the CSS, control, and amended soil. The dashed lines indicate critical RAC values at low risk, medium risk, and high risk, respectively.

The RAC for Cr, Cu, Pb, and Zn in the CSS, control, and amended soil is shown in Figure 4. The CSS showed no risk for Cr, Cu, and Zn, and a low risk for Pb. The control showed no risk for Cr, Cu, and Pb, and a low risk for Zn. The addition of CSS only resulted in a conspicuous increase in the RAC values of Zn, with a medium risk for Zn in all amended soils. The RAC value of Zn was increased with CSS addition rate. Overall, the medium risk of Zn indicated a substantial risk of Zn mobilisation in the amended soil.

The noncarcinogenic and carcinogenic risks for Cr, Cu, Pb, and Zn in the control and amended soil via incidental ingestion are tabulated in Table 5. For noncarcinogenic risk, the HQ values of these four elements and HI values for children and adults were increased with CSS addition rate. The HI values for children and adults in the soil amended with 25% CSS were approximately 3.7 times higher than in the control. Nevertheless, both HI values were lower than the safe level (=1), indicating negligible noncarcinogenic risks from Cr, Cu, Pb, and Zn in the amended soil for children and adults. For carcinogenic effects, the carcinogenic risk levels of Cr for children and adults were increased with CSS addition rate. The addition of CSS at a rate of 25% resulted in a large increase in the carcinogenic risk levels of Cr for children and adults by approximately 12 times, compared with the control. However, both carcinogenic risk levels of Cr were below the acceptable limit (=1 × 10^−4^), suggesting that the carcinogenic risk of Cr in the amended soil can be acceptable.

## 4. Conclusions

The addition of CSS significantly altered the quality of the urban garden soil selected for this study, from the viewpoint of total heavy metal concentration, fractionation, and bioaccessibility. The total concentrations, nonresidual fractions, and bioaccessibility of Cr, Cu, Pb, and Zn were increased with CSS addition rate. This can be ascribed to the relatively high concentrations of these heavy metals and their distinct chemical forms in the CSS, as well as changes in soil properties, such as pH and OM content, which can influence metal retention by the amended soil. The Cr, Cu, and Zn bioaccessibility correlated positively with the total concentration, while the Cr, Cu, Pb, and Zn bioaccessibility correlated negatively with the residual fraction. Exchangeable/residual and bioaccessible fractions are used as the key indicators of potential risks which metals pose to the environment and human health, while total concentration may overestimate the risk. As demonstrated in this study, the risk assessment incorporating fractionation (viz. RAC analysis) and oral bioaccessibility (viz. HI and cancer risk calculation) can provide a more accurate assessment of the environmental and health risks for heavy metals in the CSS-amended soil. Although the use of biosolids in landscaping can restrain the adverse effect of heavy metals in biosolids on food production, it is advisable that in green areas of cities amended with biosolids, it is of great importance to know the mobility and bioaccessibility of heavy metals in the amended soil. In addition, future field investigation on the long-term effect of biosolids on heavy metal behaviour in the soils of urban recreational areas is needed, because soil properties (e.g., pH and OM content) tend to be influenced by human activities and the environment. 

## Figures and Tables

**Table 1:** Physicochemical properties of the garden soil and composted sewage sludge (CSS) in the present study and of urban park soils in Jinan, Shandong Province. EC: electrical conductivity; CEC: cation exchange capacity; OM: organic matter; TN: total nitrogen.

Item	Garden Soil ^a^	CSS ^a^	Urban Park Soils in Jinan ^b^
pH_1:5 solid/water_	8.46 ± 0.05	7.58 ± 0.02	8.26 ± 0.19
EC_1:5 solid/water_ (μS/cm)	142 ± 18	5013 ± 103	-
CEC (cmol(+)/kg)	15.3 ± 0.6	45.4 ± 0.8	-
OM (%)	0.64 ± 0.00	25.63 ± 0.53	1.49 ± 0.91
TN (%)	0.04 ± 0.00	1.20 ± 0.03	0.09 ± 0.04
Clay (0.01–2 μm *v*/*v* %)	12.2 ± 0.4	6.0 ± 0.3	-
Silt (2–20 μm *v*/*v* %)	34.7 ± 0.3	32.5 ± 1.6	-
Sand (20–2000 μm *v*/*v* %)	53.1 ± 0.6	61.5 ± 1.9	-

^a^ Data were expressed as mean ± standard deviation (*n* = 3). ^b^ A total of 55 composite soil samples (0–0.4 m) were collected from 12 main urban parks in Jinan. Data were expressed as mean ± standard deviation (*n* = 55) [32].

**Table 2:** Total heavy metal concentrations in the control and amended soil.

CSS Addition Rate (%)	Cr (mg/kg)	Cu (mg/kg)	Pb (mg/kg)	Zn (mg/kg)
Control	70.5 ± 5.0 a	24.0 ± 2.8 a	28.7 ± 2.4 a	73.5 ± 5.0 a
5	78.0 ± 6.0 ab	36.3 ± 1.2 b	30.1 ± 1.2 a	91.0 ± 10.4 ab
10	81.7 ± 4.0 bc	48.0 ± 2.7 c	36.6 ± 7.3 ab	106.7 ± 8.0 b
25	89.0 ± 3.6 c	79.7 ± 9.0 d	39.2 ± 5.3 b	149.7 ± 7.5 c

Data were expressed as mean ± standard deviation (*n* = 3). For a given element, the values labelled with the same letter were not significantly different at a significance level of *p* < 0.05 by the least significant difference (LSD) test.

**Table 3:** Ceiling concentrations of the selected heavy metals in the sewage sludge quality standard for landscaping in China, soil quality standards in China and Australia, and soil background values in Shandong Province.

Element	Ceiling Concentrations (mg/kg)						Soil Background Values in Shandong Province (mg/kg) ^d^
	Sludge Quality Standard	Soil Quality Standard					
	China ^a^	China ^b^			Australia ^c^		
	Soil pH ≥ 6.5	Grade A	Grade B (pH > 7.5)	Grade C	HIL A	HIL C	
Cr	1000	90	250	300	100	300	64.3
Cu	1500	35	100	400	6000	17,000	22.3
Pb	1000	35	350	500	300	600	24.5
Zn	4000	100	300	500	7400	30,000	60.9

^a^ Reference values were reported by the General Administration of Quality Supervision, Inspection, and Quarantine and the Standardization Administration [33]. ^b^ Reference values were reported by the former National Environmental Protection Agency [34]. Grade A includes contaminant upper limits to protect regional natural ecology. Grade B includes contaminant upper limits to ensure agricultural production and human health. Grade C includes contaminant upper limits to ensure agricultural production and plant growth. ^c^ Reference values were reported in the National Environment Protection (Assessment of Site Contamination) Measure 1999 [35]. Health investigation level (HIL) A: Residential with garden/accessible soil (where home-grown produce is <10% fruit and vegetable intake (no poultry)); also includes childcare centres, preschools, and primary schools. HIL C: Public open space such as parks, playgrounds, playing fields (e.g., ovals), secondary schools, and footpaths. ^d^ Reference values were reported by the former National Environmental Protection Agency and China National Environmental Monitoring Centre [36].

**Table 4:** Correlation coefficients (*r*) between heavy metal bioaccessibility and total concentration, residual fraction, and physicochemical properties in the control and amended soil (*n* = 12).

Metal_Bioaccessibility_	Metal_Total_	Metal_Residual_	pH	EC	CEC	OM
Cr_Bioaccessibility_	0.840 **	−0.985 **	−0.956 **	0.989 **	0.976 **	0.965 **
Cu_Bioaccessibility_	0.842 **	−0.788 **	−0.957 **	0.919 **	0.905 **	0.849 **
Pb_Bioaccessibility_	−0.025	−0.646 *	−0.532	0.512	0.494	0.520
Zn_Bioaccessibility_	0.905 **	−0.919 **	−0.957 **	0.977 **	0.926 **	0.910 **

* Significance level of *p* < 0.05. ** Significance level of *p* < 0.01.

**Table 5:** Health risks from exposure to heavy metals in the control and amended soil via incidental ingestion. HQ: hazard quotient. HI: hazard index.

CSS Addition Rate (%)	Receptor	Cr		Cu	Pb	Zn	HI
		HQ	Carcinogenic Risk	HQ	HQ	HQ	
Control	Children	2.16 × 10^−3^	2.78 × 10^−7^	5.33 × 10^−4^	1.26 × 10^−2^	3.14 × 10^−4^	1.56 × 10^−2^
	Adults	2.32 × 10^−4^	1.19 × 10^−7^	5.71 × 10^−5^	1.35 × 10^−3^	3.37 × 10^−5^	1.67 × 10^−3^
5	Children	8.72 × 10^−3^	1.12 × 10^−6^	1.97 × 10^−3^	1.75 × 10^−2^	7.34 × 10^−4^	2.89 × 10^−2^
	Adults	9.34 × 10^−4^	4.80 × 10^−7^	2.11 × 10^−4^	1.87 × 10^−3^	7.86 × 10^−5^	3.10 × 10^−3^
10	Children	1.33 × 10^−2^	1.70 × 10^−6^	3.12 × 10^−3^	1.77 × 10^−2^	1.22 × 10^−3^	3.53 × 10^−2^
	Adults	1.42 × 10^−3^	7.30 × 10^−7^	3.35 × 10^−4^	1.90 × 10^−3^	1.31 × 10^−4^	3.79 × 10^−3^
25	Children	2.56 × 10^−2^	3.29 × 10^−6^	6.33 × 10^−3^	2.40 × 10^−2^	2.28 × 10^−3^	5.82 × 10^−2^
	Adults	2.74 × 10^−3^	1.41 × 10^−6^	6.78 × 10^−4^	2.57 × 10^−3^	2.44 × 10^−4^	6.23 × 10^−3^
